# Development of an Influenza A Master Virus for Generating High-Growth Reassortants for A/Anhui/1/2013(H7N9) Vaccine Production in Qualified MDCK Cells

**DOI:** 10.1371/journal.pone.0160040

**Published:** 2016-07-25

**Authors:** Yasushi Suzuki, Takato Odagiri, Masato Tashiro, Eri Nobusawa

**Affiliations:** Influenza Virus Research Center, National Institute of Infectious Diseases, Musashi-murayama, Tokyo, Japan; University of Rochester Medical Center, UNITED STATES

## Abstract

In 2013, the first case of human infection with an avian influenza A virus (H7N9) was reported in China, and the human infection with this virus has continued as of 2016. At the request of the WHO, we have successfully developed candidate reassortant vaccine virus using A/Anhui/1/2013 and the high egg-growth master virus A/PR/8/1934. Recent plans regarding influenza vaccine production include using cell-cultured systems in Japan and several other countries. However, egg-based vaccine viruses are not always suitable for cell-cultured vaccine production due to potential issues with growth, protein yield and antigenic stability. Therefore, in this study, we have developed a high-growth master virus (hg-PR8) adapted to qualified NIID-MDCK cells that are competent for vaccine production. The virus hg-PR8 was obtained after 20 serial passages of A/Puerto Rico/8/1934 (PR8) in NIID-MDCK cells. The viral titer of hg-PR8 was 10^8.6^ plaque-forming units per milliliter (PFU/mL). Seven amino acid substitutions were identified in the amino acid sequences of PB2, PB1, PA, NA, M and NS of hg-PR8 compared to the sequence of the original PR8 (org-PR8) strain. The growth capacities of the reassortant viruses, which possess heterologous internal genes from hg-PR8 or org-PR8, indicated that the amino acid changes in PB2 and NS2 similarly affected growth capacity in NIID-MDCK cells. To assess the suitability of hg-PR8 as a master virus, we generated 6:2 reassortant viruses possessing the HA and NA segments from A/Anhui/1/2013 (H7N9) and the remaining segments from hg-PR8. The virus titers of the reassortant strains were 10^7^−10^8^ PFU/mL. The antigenicity of the viruses was stable during ten passages of the viruses in NIID-MDCK cells. In comparison with the egg-based reassortant vaccine viruses with identical HA and NA segments, the hg-PR8-based viruses showed 1.5- to 2-fold higher protein yields in NIID-MDCK cells.

## Introduction

Influenza vaccination is the primary strategy for influenza prevention and control, and it has been used for more than 60 years. Influenza vaccines are effective primarily in preventing the elderly and high-risk patients from experiencing the severe outcomes of influenza [1]. Currently, viruses for influenza vaccine production have been propagated primarily in embryonated chicken eggs. However, the circulating human influenza viruses do not always grow well in eggs [2]. Therefore, reassortant viruses generated from the circulating viruses and an egg-adapted high-growth virus, such as A/Puerto Rico/8/1934 (PR8), have been utilized as seasonal influenza vaccine viruses [3].

Recently, frequent amino acid substitutions in hemagglutinin (HA) have been reported during the propagation of the viruses in eggs [4–9]. These egg-adapted mutations often influence the receptor specificity and the antigenicity of the viruses. Such mutations in vaccine viruses may reduce the efficacy of the influenza vaccines [10]. To avoid these antigenic changes in vaccine viruses, propagation of these viruses in mammalian cell lines instead of in eggs has been suggested. In this case, characterization and qualification of the cell lines as bioreactors for primary virus isolation and propagation of candidate vaccine viruses is required to ensure their safety. Furthermore, the antigenic stability and antigen yields of the vaccine viruses should be considered. In this context, Donis et al. investigated the characteristics of the cell lines qualified for influenza virus isolation and propagation in terms of their cell-cultured influenza vaccine production [11]. Several groups have taken alternative approach of attempting to improve the virus growth and protein yields of the cell-cultured vaccine viruses, i.e., development of a high-growth master virus for creating reassortant viruses and genetic modification of the viral genome to improve the virus growth and/or yields [12–16].

In April 2013, the first three fatal cases of human infection with a low pathogenic avian influenza A virus (H7N9) in China were reported. Since then, until 2016, human infection with this virus has continued in China [17]. Because of the pandemic threat posed by this virus, we have developed egg-grown candidate 6:2 reassortant vaccine viruses using reverse genetics (RG) techniques at the request of the World Health Organization (WHO) [18]. Incidentally, egg-based influenza vaccines production is planned to switch to cell-cultured vaccine production in Japan and other countries. Cell-cultured vaccines are preferable particularly for pandemic vaccines because of independence from huge amount of egg supply [19]. Currently cell-cultured vaccine production has been performed in, Madin Darby canine kidney (MDCK), African Green Monkey Kidney (Vero), and EB66 cells [20–22] using vaccine viruses developed for vaccine production in embryonated chicken eggs. However, viruses typically cultured in eggs may not grow as well in the cultured cells. Therefore, the generation of reassortant vaccine viruses using an established master virus that is adapted to the qualified cells would be a great improvement in vaccine production. In this context, we have developed a high-growth master virus, hg-PR8, which is adapted to the NIID-MDCK qualified cell line. Using hg-PR8, we have successfully generated cell-cultured 6:2 reassortant vaccine viruses for H7N9 influenza. We then compared the growth capacities and viral protein yields of these viruses with their counterparts, egg-growth vaccine viruses.

## Materials and Methods

### Cells and Viruses

The NIID-MDCK cells were derived from MDCK cells from the American Type Culture Collection (Rockville, MD, USA). The original MDCK cells were adapted to serum-free medium by continuous culturing in Opti-Pro SFM (GIBCO, Carlsbad, CA, USA) containing 2 mM L-glutamine. The resultant cells, NIID-MDCK cells, were approved to meet the standard for vaccine production in compliance testing at BioReliance (Glasgow, UK). A human embryonic kidney cell line, 293T, was cultured in DMEM (GIBCO) supplemented with 10% fetal bovine serum (FBS). All cells were maintained under 5% CO_2_ at 37°C. Egg-grown viruses of the original PR8 virus (org-PR8), H7N9 viruses (A/Anhui/1/2013 and A/Shanghai/1/2013), A/mallard/Netherlands/12/2000(H7N3) and two H7N9 reassortants, NIIDRG-10 and NIIDRG-10.1 [18], were used in this study. The HA and NA genes of the latter two viruses are identical to those of the WHO-approved egg-based vaccine viruses A/Anhui/1/2013 (NIBRG-268) and A/Anhui/1/2013 (NIIDRG-10.1), respectively. The original virus stock of A/Anhui/1/2013 exhibited minor heterogeneity, including distinguishable viruses with different HA amino acid sequences [18]. NIBRG-268/NIIDRG-10 and NIIDRG-10.1 have the HA with the different amino acid sequences of the original A/Anhui/1/2013 [18].

### Adaptation of PR8 virus to the NIID-MDCK cells

The org-PR8 virus was propagated in NIID-MDCK cells at a 10-fold serial dilution with the infection medium (MEM supplemented with 0.4% bovine serum albumin, 100 μg/mL of kanamycin, and 2 μg/mL of TPCK-trypsin) under 5% CO_2_ at 34°C. At 72 hours post-infection (hpi), the supernatant was collected from the highest dilution that showed hemagglutination activity and was used for the next passage. In this way, ten serial passages of the PR8 virus in NIID-MDCK cells were performed. The viruses at passage 10 were purified through five additional rounds of plaque assays, including plaque picking and inoculation at a 10-fold serial dilution in NIID-MDCK cells. Virus titers were determined in a plaque assay using infection medium containing 0.8% agarose. Genetic analysis of the viruses was performed using the Sanger sequencing methods.

### Viral growth kinetics in NIID-MDCK cells

NIID-MDCK cells were infected with viruses at a multiplicity of infection (m.o.i.) of 0.0001 and were incubated with the infection medium at 34°C under 5% CO_2_. Then, supernatants were collected at 24, 48, and 72 hpi and stored at -80°C. The virus titers at each time point were determined by a plaque assay [23]. Because the plaques were detected more easily at 4 days p.i. than at 3 days p.i., the cells were fixed and stained with 0.1% crystal violet solution in 20% ethanol at 4 days p.i. The number of plaques was used to calculate the plaque forming units (PFU/mL) of each virus.

### Plasmid construction

The eight gene segments of the PR8 virus were amplified by reverse transcription and PCR using primers specific for each gene. Each segment was then cloned into RNA polymerase I-driven plasmids (pPolI) [24].

### Generation of viruses using RG technique

Rg-hg-PR8 and reassortant viruses were generated using a twelve plasmid-based RG technique as previously described [24]. In brief, eight pPolI plasmids encoding the PB2, PB1, PA, HA, NP, NA, M, and NS genes and four pCAGGS plasmids encoding the PB2, PB1, PA and NP proteins were co-transfected into 293T cells using the Trans^®^IT-LT1 Transfection Reagent (Mirus, Madison, WI, USA) as described previously [18]. To create the two 6:2 reassortant viruses, NIIDRG-10C and -10.1C, we co-transfected two pPolI plasmids encoding the HA and NA genes of NIIDRG-10 and NIIDRG-10.1 [18] and six pPolI plasmids encoding six internal proteins of hg-PR8 together with the four pCAGGS plasmids into 293T cells (Table 1). The supernatant was collected at 48 h post-transfection and was then inoculated into NIID-MDCK cells in infection medium.

**Table 1:** The gene constellation of the 6:2 reassortant viruses created in this study.

Virus	Viral origin of each segment			Reference
	HA	NA	Other segments	
NIIDRG-10C	Anhui/1^a^ (EPI439507^b^)	Anhui/1 (EPI439509)	hg-PR8	This study
NIIDRG-10	Anhui/1 (EPI439507)	Anhui/1 (EPI439509)	Egg-grown PR8	Nakamura et al. [18]
NIIDRG-10.1C	Anhui/1 (EPI486492)	Anhui/1 (EPI439509)	hg-PR8	This study
NIIDRG-10.1	Anhui/1 (EPI486492)	Anhui/1 (EPI439509)	Egg-grown PR8	Nakamura et al. [18]

^a^ Anhui/1 = A/Anhui/1/2013(H7N9).

^b^ Accession number of Global Initiative on Sharing Avian Influenza Data (GISAID EpiFlu database) for each gene.

### Hemagglutination-inhibition (HAI) assay

Antigenic characterizations of reassortant viruses were performed using hemagglutination inhibition (HAI) test with 0.5% turkey erythrocytes [25]. Post-infection ferret antisera against A/Anhui/1/2013 (H7N9), NIIDRG-10.1, A/Shanghai/1/2013 (H7N9), and A/mallard/Netherlands/12/2000 (H7N3) were used for the HAI tests.

### Measurement of total viral proteins

NIID-MDCK cells were infected with viruses at an m.o.i. of 0.001 and were incubated at 34°C under 5% CO_2_. At 72 hpi, the supernatants were harvested and treated with ß-propiolactone (0.05%) for 16 h at 4°C to inactivate the viruses. Inactivated viruses were purified by ultracentrifugation through 20% sucrose at 112,500 g for 90 min at 4°C using an Optima L-90K ultracentrifuge with a SW28 rotor (Beckman Coulter Inc.). The pellets were rinsed with PBS, concentrated by ultracentrifugation as mentioned above and suspended in an appropriate volume of PBS. The protein amount of the virus concentrate was determined using Micro BCA Protein Assay Kit (Thermo, Rockford, IL, USA) according to the manufacturer’s instructions.

### Western blotting

The virus concentrates were analyzed by SDS-PAGE on 12.5% gels. After electrophoresis, the viral proteins were transferred to polyvinylidene difluoride (PVDF) membranes and detected by a rabbit polyclonal antibody against recombinant HA protein of H7N9 (A/Shanghai/1/2013) (Sino Biological Inc. Beijing, China) and a donkey anti-rabbit IgG antibody conjugated with horseradish peroxidase (GE Healthcare, UK Ltd., Little Chalfont, England) as the secondary antibody. When necessary, the virus concentrates were denatured for 10 min at 100°C and treated with 1 U PNGase F (New England BioLabs Inc., Ipswich, MA, USA) per μg of protein for 16 h at 37°C.

## Results

### Development of a high-growth master virus

**Fig 1:**
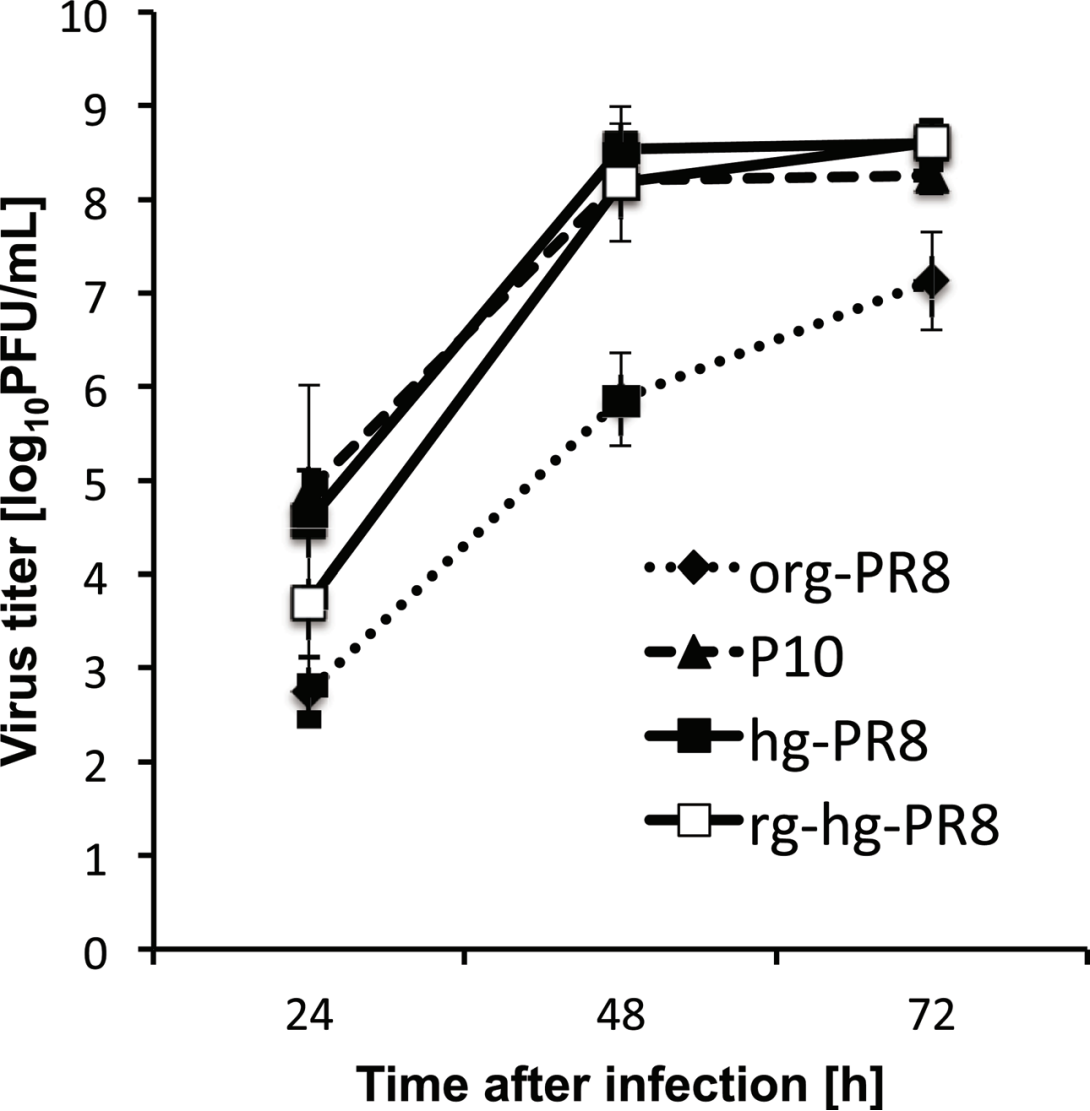
Growth capacities of PR8 viruses in NIID-MDCK cells. Replication kinetics of original PR8 (org-PR8), the passage-10 virus (P10), hg-PR8 and rg-hg-PR8 were compared in NIID-MDCK cells. NIID-MDCK cells were inoculated with the respective viruses at an m.o.i. of 0.0001. The supernatants were collected at 24, 48, and 72 hpi, and the virus titers were determined in a plaque formation assay. The vertical axis shows the mean PFUs ± SD at each time point for three or more independent experiments.

**Fig 2:**
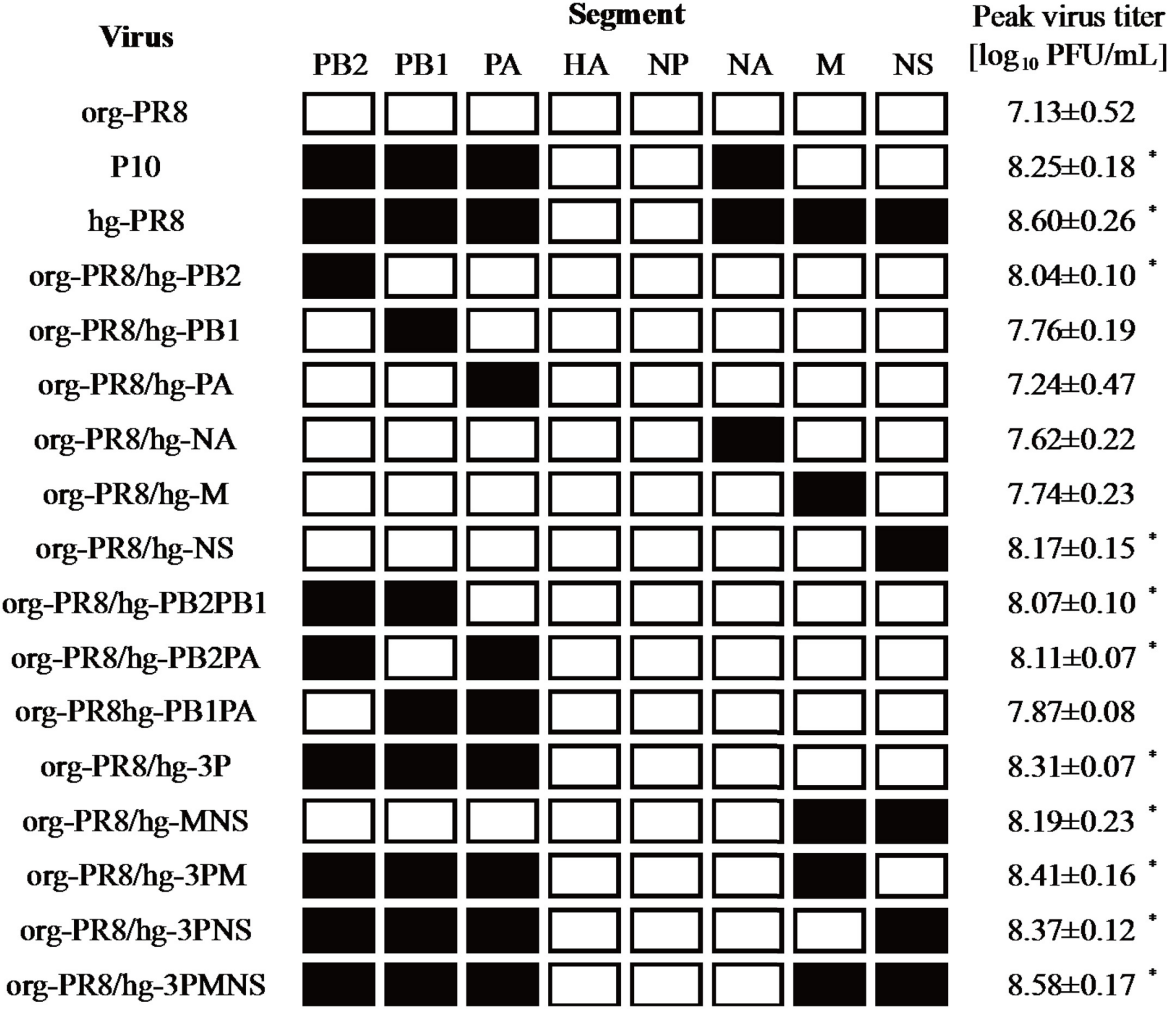
Gene constellations and the growth capacities of the reassortants between org-PR8 and hg-PR8 viruses. The gene constellations of org-PR8, P10, hg-PR8 and reassortant viruses between org-PR8 and hg-PR8 are indicated by black and white squares. Black squares indicate gene segments containing non-synonymous mutations compared with the sequences of the corresponding segment of org-PR8. NIID-MDCK cells were inoculated with these viruses at an m.o.i. of 0.0001. The peak virus titers were determined in a plaque formation assay. Significant differences in the peak virus titers were assessed with Bonferroni’s multiple-comparison test. (*p<0.*01* from org-PR8)

To develop a master virus to generate high-growth reassortant viruses in qualified cultured cells, we adapted a laboratory strain of PR8 to NIID-MDCK cells. The org-PR8 virus with a virus titer of 10^7.1^ PFU/mL was subjected to multiple passages in NIID-MDCK cells as described in the Materials and Methods. As a result, the virus titer increased to 10^8.3^ PFU/mL at 72 hpi after ten serial passages of the org-PR8 viruses in NIID-MDCK cells (Fig 1). Then, we repeated plaque purification and propagation of the virus in NIID-MDCK cells an additional five times as described in the Materials and Methods. Finally, we obtained a high-growth PR8 virus (hg-PR8) with an infectivity titer of 10^8.6^ PFU/mL at 72 hpi (Fig 1). The peak titers of PR8 virus at passage 10 in NIID-MDCK cells and of hg-PR8 were significantly higher than that of the org-PR8 (*p<0*.*01*), but no considerable difference was observed in the titers between passage-10 and hg-PR8 viruses. To understand the reason for the high growth capacity of hg-PR8, we compared the entire nucleotide sequences of the eight gene segments between the org-PR8 and hg-PR8 viruses. The deduced amino acid sequences indicated that seven amino acid substitutions (D701N in PB2, H486N in PB1, V44I and G66D in PA, K247R in NA, A137T in M1, and S25L in NS2) had occurred during the ten passages of the org-PR8 virus in NIID-MDCK cells. All of the amino acid substitutions except those in M1 and NS2 appeared at the passage 10 (Table 2). In contrast, no amino acid change was noted in HA during the passages. To identify the mutations responsible for the high growth capacity of the hg-PR8 virus, we created genetic reassortants between the org-PR8 and hg-PR8 viruses and compared their growth properties (Fig 2).

**Table 2:** Amino acid substitutions during passages of the original PR8 virus in NIID-MDCK cells.

Segment	Amino acid position	Amino acid residue at each position of the respective virus		
		original PR8	Passage-10	hg-PR8
PB2	701	D	N	N
PB1	456	H	N	N
PA	44	V	I	I
PA	66	G	D	D
NA	247	K	R	R
M1	137	A	A	T
NS2	25	S	S	L

The org-PR8/hg-3P virus exhibited a peak virus titer similar to that of P10, and org-PR8/hg-3PMNS exhibited a peak virus titer comparable to that of hg-PR8, suggesting that the mutation in NA of P10 has little effect on growth ability (Fig 2). The peak virus titers of the reassortants org-PR8/hg-PB2 and org-PR8/hg-NS were significantly higher than that of org-PR8 (*p<0*.*01*; Fig 2). Other reassortants that possessed the hg-PB2 or hg-NS segments also exhibited significantly higher peak virus titers than org-PR8 (*p<0*.*01*; Fig 2). These results suggest that the D701N substitution in PB2 and the S25L substitution in NS2 were mainly responsible for the increased growth capacity of hg-PR8.

To assess genetic suitability of the hg-PR8 virus, which is required as a master virus for preparation of candidate vaccine viruses to be used for vaccine production in tissue culture cell systems, we generated reassortants between hg-PR8 and wild-type viruses.

### Generation of the 6:2 reassortant viruses between A/Anhui/1/2013 and hg-PR8

Two 6:2 reassortant viruses between A/Anhui/1/2013 and hg-PR8, i.e., NIIDRG-10C and NIIDRG-10.1C, were created using the RG technique as described in the Materials and Methods. The nucleotide sequences of the HA and NA genes of the respective viruses were identical to those of egg-grown 6:2 reassortants NIIDRG-10 and 10.1 (Table 1) [18]. The amino acid residue at position 125 of HA (H7 numbering for mature HA) was alanine and threonine in NIIDRG-10C and NIIDRG-10.1C, respectively. In our previous study, this difference in the amino acid residue at position 125 affected the growth capacity of NIIDRG-10 and NIIDRG-10.1 [18].

### Growth properties of 6:2 reassortant viruses

**Fig 3:**
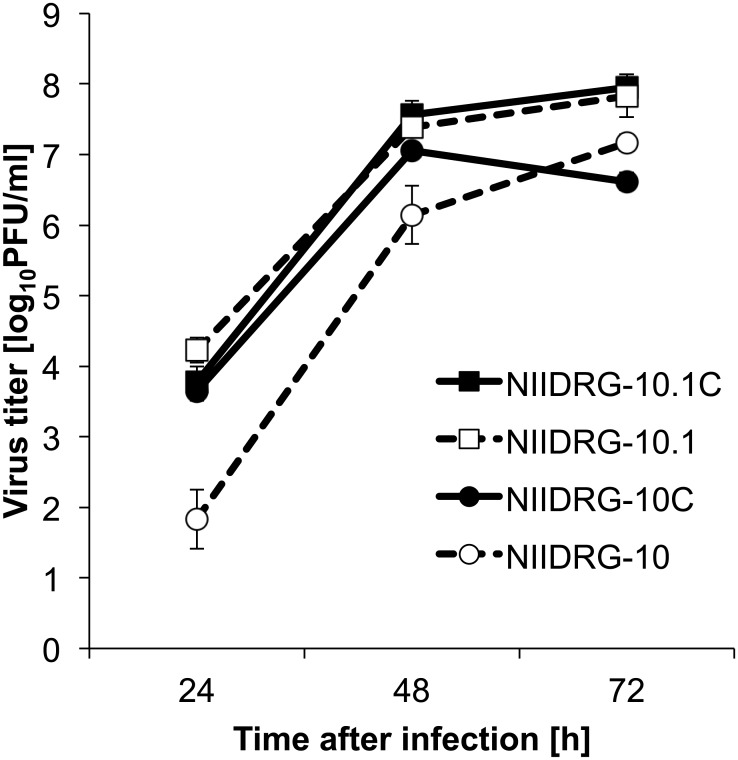
Comparison of the growth capacities between the reassortants in NIID-MDCK cells. The growth kinetics of NIIDRG-10.1C, NIIDRG-10.1, NIIDRG-10C and NIIDRG-10 in NIID-MDCK cells were determined. NIID-MDCK cells were inoculated with the respective viruses at an m.o.i. of 0.0001. The supernatants were collected at 24, 48, and 72 hpi, and the virus titers were determined in a plaque formation assay. The vertical axis shows the mean PFUs ± SD at each time point (N = 3).

We then examined whether the differences in the master viruses influenced on the viral growth capacity in NIID-MDCK cells. NIID-MDCK cells were infected with NIIDRG-10C, NIIDRG-10.1C or rg-hg-PR8 created by the RG technique. At 72 hpi, the virus titers of NIIDRG-10C and NIIDRG-10.1C were 10^6.6^ PFU/mL and 10^8.0^ PFU/mL (Fig 3), respectively, whereas the titer of rg-hg-PR8 was 10^8.6^ PFU/mL (Fig 1). The highest virus titer of NIIDRG-10C was 10^7.1^ PFU/mL at 48 hpi (Fig 3). The effect of T125 on increased virus growth of NIIDRG-10.1 in eggs was retained with NIIDRG-10.1C, regardless of the differences in the host cells.

By contrast, the egg-based reassortants NIIDRG-10 and NIIDRG-10.1 exhibited only moderately lower growth capacities in NIID-MDCK cells of 10^7.2^ PFU/mL and 10^7.8^ PFU/mL, respectively (Fig 3), than in NIIDRG-10C and NIIDRG-10.1C. These results indicated that the growth capacities of the reassortant viruses were not significantly affected by the difference in the master virus used.

### Total protein yields (TPYs) of reassortant viruses

**Fig 4:**
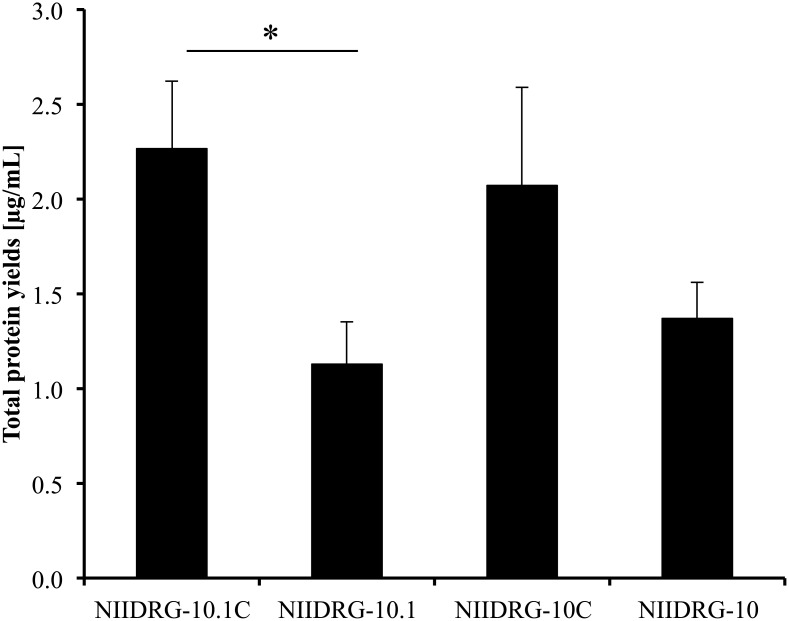
Total protein yields of reassortant viruses. Average total protein yields (TPYs) of the reassortants. The vertical axis shows the average yields ± SD for three independent experiments. The differences in the yields between the reassortants were statistically analyzed using Welch’s t-test. (*p<0.*05*)

To understand the effect of the difference in master virus on TPYs, we compared the TPYs of NIIDRG-10.1C, NIIDRG-10.1, NIIDRG-10C, and NIIDRG-10 as described in the Materials and Methods. The TPYs of NIIDRG-10.1C and NIIDRG-10C were 2.3 μg/mL and 2.1 μg/mL, while those of NIIDRG-10.1 and NIIDRG-10 were 1.1 μg/mL and 1.4 μg/mL, respectively (Fig 4). The TPY of NIIDRG-10.1C was significantly higher than that of NIIDRG-10.1 (*p<0*.*05*). The TPY of NIIDRG-10C was slightly higher than that of NIIDRG-10. Although the amino acid sequences of HA and NA from NIIDRG-10C and NIIDRG-10.1C were comparable with those of NIIDRG-10 and NIIDRG-10.1, respectively, the TPYs in the cell-based reassortant viruses were 1.5 to 2 times higher than those of the egg-based reassortant viruses, suggesting that the difference(s) in the master viruses influenced TPYs of their reassortant viruses.

### N-linked glycosylation of NIIDRG-10.1C HA

**Fig 5:**
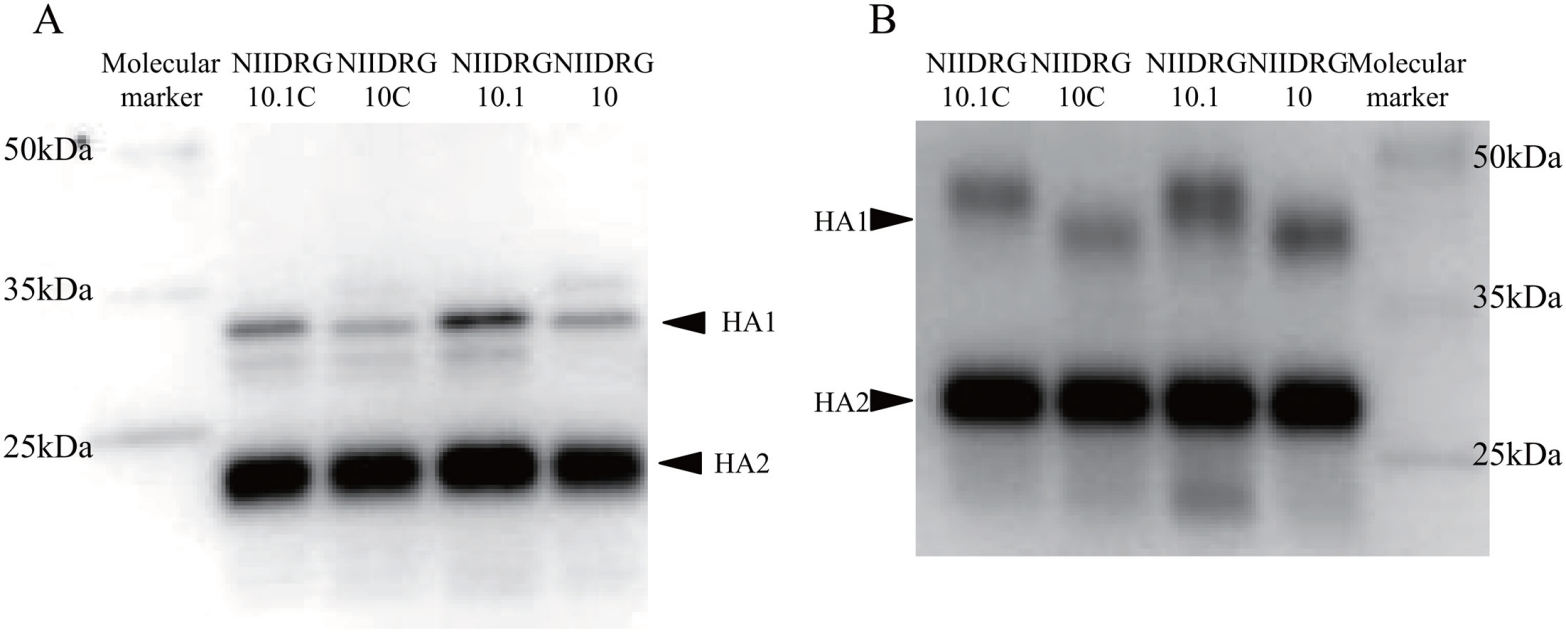
Western blotting analysis of the purified viral proteins. Purified viral concentrates of NIIDRG-10C, -10.1C, -10 and -10.1 were analyzed by SDS-PAGE. HA proteins were detected using a rabbit polyclonal antibody against recombinant HA protein of H7N9 (A/Shanghai/1/2013) (Sino Biological Inc. Beijing, China) and a donkey anti-rabbit IgG horseradish peroxidase-conjugated secondary antibody by western blotting analysis. Purified viral proteins were treated (A) or untreated (B) with N-glycosidase F.

In a previous study, we observed an additional glycosylation at position N123 in the HA of NIIDRG-10.1 due to the amino acid substitution A125T [18]. To confirm that this additional N-linked glycosylation also occurred in the HA of NIIDRG-10.1C, we compared the electrophoretic mobilities of the HA proteins among NIIDRG-10C, NIIDRG-10.1C, NIIDRG-10, and NIIDRG-10.1 viruses. Purified virus concentrates were treated with or without PNGase F and analyzed by western blotting. The HA protein of each virus showed similar mobility to one another if they were treated with PNGase F, while the HA proteins from NIIDRG-10.1C and NIIDRG-10.1 migrated more slowly than the other HA proteins without the PNGase F treatment (Fig 5). These results suggested that an additional N-linked glycosylation had also occurred at position 123 in the HA proteins of NIIDRG-10.1 and NIIDRG-10.1C.

### Multiple passages of NIIDRG-10.1C and NIIDRG-10C in NIID-MDCK cells

To assess the genetic stability of the HA and NA genes, NIIDRG-10.1C and NIIDRG-10C were passaged ten times in NIID-MDCK cells. After the 10th passage, while a mutation for the amino acid change N239D was detected in the HA gene from NIIDRG-10C, no mutation was observed in the HA and NA genes of NIIDRG-10.1C. In our previous study, multiple passages of NIIDRG-10.1 in chicken embryonated eggs resulted in the appearance of amino acid substitutions in HA at egg passage 7. These amino acid substitutions induced a 4-fold reduction in the HI titer of the ferret antisera against NIIDRG-10.1 [18]. Thus, NIIDRG-10.1C exhibits genetic and antigenic stability in NIID-MDCK cells.

By contrast, NIIDRG-10C exhibited heterogeneous residues N/D at position 239 in the HA at cell passage 5, and D239 became predominant in passages 6 to 10. No nucleotide mutation was found in the NA gene of NIIDRG-10C at passage 10.

### Antigenic analysis of NIIDRG-10.1C and NIIDRG-10C

To determine the antigenicity of NIIDRG-10.1C and NIIDRG-10C, HAI test was performed using ferret antisera against the original egg isolates of A/Anhui/1/2013 (H7N9), A/Shanghai/1/2013 (H7N9) and A/mallard/Netherlands/12/2000 (H7N3) (Table 3). The antiserum against A/Anhui/1/2013 virus reacted well with NIIDRG-10C and NIIDRG-10.1C, suggesting that both viruses retained a similar antigenicity to A/Anhui/1/ 2013. The antisera against other H7 subtype viruses also reacted with the NIIDRG-10C and NIIDRG-10.1C, with HAI titers within a 4-fold range of those against the homologous viruses. The reactivity of these antisera with NIIDRG-10C and NIIDRG-10.1C was similar to that of NIIDRG-10 and NIIDRG-10.1 in our previous study [18]. However, the HI titers of these antisera against NIIDRG-10C and NIIDRG-10.1C differed by 4-fold. This difference in reactivity is probably due to the presence or absence of N-glycosylation at 123N of HA.

**Table 3:** Antigenic characterization of NIIDRG-10.1C and NIIDRG-10C by HAI test.

Virus	HAI titers of each ferret antiserum raised against the virus^a^			
	A/Anhui/1/2013 (H7N9)	A/Shanghai/1/2013 (H7N9)	A/mallard/ Netherlands/12/2000 (H7N3)	NIIDRG-10.1
A/Anhui/1/2013 (H7N9)	**80**^b^	160	160	80
A/Shanghai/1/2013(H7N9)	20	**160**	160	20
A/mallard/Netherlands/12/2000 (H7N3)	20	80	**80**	20
NIIDRG-10.1	40	80	80	**80**
NIIDRG-10.1C	40	80	80	80
NIIDRG-10C	160	320	320	160
NIIDRG-10C- N239D	160	320	320	160

^a^ Each virus was propagated in embryonated chicken eggs.

^b^ Bold indicates HAI titer for the homologous strain.

The antiserum against NIIDRG-10.1 reacted well with NIIDRG-10.1C and NIIDRG-10C (Table 3). NIIDRG-10C at passage 6 was also recognized well by those antisera despite the N239D substitution, suggesting that this amino acid change did not affect the antigenicity of NIIDRG-10C.

## Discussion

In this study, we have isolated hg-PR8 virus by passaging the org-PR8 virus in NIID-MDCK cells (Table 2 and Fig 1). To assess the suitability of hg-PR8 as a master virus for generating vaccine viruses, two 6:2 reassortant viruses between A/Anhui/1/2013 (H7N9) and hg-PR8 were created. Both two reassortant viruses exhibited high growth capacity in NIID-MDCK cells (Fig 3).

The hg-PR8 virus had seven amino acid substitutions in the internal proteins and NA protein, compared to the corresponding amino acid residues of org-PR8 virus (Table 2). The D701N substitution in PB2 is known to improve PB2’s binding capacity to mammalian importin α [26, 27]. In this context, other groups have reported that the D701N substitution enhanced virus replication of H5N1 and H1N1pdm09 in mouse epithelial cells or human lung epithelial cells and increased the pathogenicity of H5N1 viruses in a mouse model [28, 29]. Our result implied that the N701 of hg-PR8 PB2 also plays a role in the improved growth capacity of the virus in NIID-MDCK cells. The interaction of hg-PR8 PB2 with importin α in NIID-MDCK cells remains to be investigated. In addition to D701N in PB2, the S25L substitution in NS2 was predominantly related to the increase in the peak virus titer of hg-PR8.

Based on the peak virus titers of the reassortants, all amino acid changes in hg-PR8 except for that in NA were likely involved in the high growth ability of hg-PR8 (Table 2). However, none of the mutations except D701N in PB2 have been reported to correlate with virus growth ability. The influence of these mutations on growth ability remains to be clarified. It is noteworthy that no amino acid substitution was found in the HA protein during the serial passages of org-PR8 in NIID-MDCK cells. This fact suggests that improvement in the growth capacity of hg-PR8 in NIID-MDCK cells was not related to the receptor-binding affinity of the HA protein.

Adaptation of influenza vaccine viruses to embryonated chicken eggs frequently causes antigenic changes to the vaccine viruses, occasionally resulting in a vaccine failure. To avoid the inconvenience, cell-cultured vaccines have been considered an alternative way to manufacture influenza vaccines. In this study, using the qualified cell-adapted hg-PR8 virus, we have developed NIIDRG-10C and NIIDRG-10.1C as the counterparts of egg-based candidate vaccine viruses NIBRG-268 and NIIDRG-10.1. After serial passages in eggs, the latter viruses acquired amino acid substitutions in the HA protein, which resulted in an antigenic change of the virus [18]. In contrast, the antigenicity of NIIDRG-10C and NIIDRG-10.1C was not altered, even after 10 passages of the viruses in NIID-MDCK cells (Table 3).

It should be noted that the egg-adapted, high-growth reassortant vaccine viruses, NIIDRG-10 and NIIDRG-10.1 [18], replicated well also in NIID-MDCK cell comparably to the cell culture-based vaccine viruses, NIIDRG-10C and NIIDRG-10.1C (Fig 3). Therefore, as far as virus growth capacity in MDCK cells, the egg-adapted reassortant vaccine viruses [18] could be used also for cell-based vaccine production in MDCK cells. However, for efficient vaccine viruses to be used in practical vaccine productions, not only virus growth capacity but also TPY of viral proteins should be considered.

Although the egg-adapted NIIDRG-10 and NIIDRG-10.1 were shown to grow well in MDCK cells (Fig 3), TPYs of these viruses remained significantly inferior to those of cell-adapted NIIDRG-10C and NIIDRG-10.1C (Fig 4). However, their counterparts, NIIDRG-10C and NIIDRG-10.1C, propagated in NIID-MDCK cells showed high TPYs similar to each other (Fig 4). With respect to the discrepancy between TPYs and growth ability, we speculate that the number of viral particles required to form one plaque may affect the TPYs. Therefore, for two viruses with similar virus titers (pfu/mL), the TPY will be higher for the virus that requires many virus particles to form one plaque than for the virus that requires fewer virus particles to form one plaque. Other groups have observed similar phenomena in which the increase in HA yields is not associated with an increase in viral titers or HA titers, such as Gomila et al. [15] and Dormitzer et al. [30]. However, further study is needed to clarify the relationship between growth capacity and TPY.

The egg-based reassortant NIIDRG-10.1 exhibited a 1.8-fold higher TPY than another egg-based reassortant, NIBRG-268 [18], possibly because of the A125T substitution in the HA protein of NIIDRG-10.1. The A125T change created an additional N-glycosylation site at position N123, which might increase the TPY of NIIDRG-10.1. In this study, we observed an additional N-glycosylation on the HA of NIIDRG-10.1C compared with NIIDRG-10C HA (Fig 5). The deduced amino acid sequence of HA was identical among NIBRG-268, NIIDRG-10 and NIIDRG-10C. However, in contrast to our initial expectation, there was no difference in TPYs between NIIDRG-10.1C and NIIDRG-10C (Fig 4).

The A125T amino acid change and/or the additional glycosylation at position 123 of HA had little effect on the TPY of NIIDRG-10.1C when the virus was propagated in NIID-MDCK cells. Although the moderately higher growth capacity of NIIDRG-10.1C than of NIIDRG-10C might be ascribed to the amino acid difference at position 125, this fact could not consist with the TPY of NIIDRG-10.1C.

However, NIIDRG-10.1C and NIIDRG-10C showed higher yields than NIIDRG-10.1 and NIIDRG-10, respectively, suggesting that hg-PR8 contributed to the difference in TPY. Regarding vaccine manufacturing efficiency, this property of hg-PR8 may be more beneficial than improvement in the viral growth capacity.

In the antigenic analysis, all antisera against H7 subtype viruses except one exhibited reactivities with NIIDRG-10C and 10.1C that differed by fourfold. We speculate that the N-glycosylation at position 123N of NIIDRG-10.1C might affect the reactivity between the antibody and HA. Such N-glycosylation presumably influences the immunogenicity of the virus. The effect of N-glycosylation at 123N of H7HA on antigenicity and immunogenicity remains to be investigated.

To date, genetic modification of the master viruses, adaptation of the viruses to Vero and MDCK cells, and changing the amino acid residues of HA have been attempted to improve the growth capacities of vaccine viruses [12–14, 31]. However, the satisfactory growth capacity of the virus does not always result in acceptable levels of TPY.

In summary, we have developed a high-growth master virus, hg-PR8, for generating cell-cultured vaccine viruses in qualified NIID-MDCK cells. Using hg-PR8, we have successfully developed cell-cultured vaccine viruses for H7N9 influenza. Based on their growth ability in NIID-MDCK cells, TPYs, antigenic identity with the original virus and antigenic stability upon multiple passages in the cells, NIIDRG-10C and NIIDRG-10.1C would be suitable as candidate vaccine viruses. In our future studies to meet the WHO approval, additional investigations into these viruses will be performed if necessary. To contribute to the stable production of cell-cultured vaccines, it may be possible to use hg-PR8 to generate other vaccine viruses in additional cultured cell lines (other MDCK, Vero and EB66 cells).
